# Role of miRNAs in Normal Endometrium and in Endometrial Disorders: Comprehensive Review

**DOI:** 10.3390/jcm10163457

**Published:** 2021-08-04

**Authors:** Kamila Kolanska, Sofiane Bendifallah, Geoffroy Canlorbe, Arsène Mekinian, Cyril Touboul, Selim Aractingi, Nathalie Chabbert-Buffet, Emile Daraï

**Affiliations:** 1 Service de Gynécologie Obstétrique et Médecine de la Reproduction, Hôpital Tenon, AP-HP, Sorbonne Université, 4 Rue de la Chine, 75020 Paris, France; sofiane.bendifallah@aphp.fr (S.B.); cyril.touboul@aphp.fr (C.T.); nathalie.chabbert-buffet@aphp.fr (N.C.-B.); emile.darai@aphp.fr (E.D.); 2 INSERM UMRS 938, Sorbonne Université, Site Saint-Antoine, 27 Rue Chaligny, CEDEX 12, 75571 Paris, France; geoffroy.canlorbe@aphp.fr (G.C.); selim.aractingi@aphp.fr (S.A.); 3 Centre Expert En Endométriose (C3E), Groupe de Recherche Clinique en Endométriose (GRC6), Sorbonne Université, 4 Rue de la Chine, 75020 Paris, France; 4 Service de Chirurgie et Cancérologie Gynécologique et Mammaire, Hôpitaux Universitaires Pitié-Salpêtrière, Charles-Foix, Sorbonne Université, 47/83, Boulevard de l’Hôpital, 75013 Paris, France; 5 Service de Médecine Interne, Hôpital Saint Antoine, AP-HP, 184 Rue du Faubourg Saint Antoine, Sorbonne Université, 75012 Paris, France; arsene.mekinian@aphp.fr; 6 Faculté de Médecine Paris 5 Descartes, 12 Rue de l’Ecole de Médecine, 75006 Paris, France

**Keywords:** miRNA, endometrium, recurrent implantation failure, endometriosis, endometrial cancer

## Abstract

The molecular responses to hormonal stimuli in the endometrium are modulated at the transcriptional and post-transcriptional stages. Any imbalance in cellular and molecular endometrial homeostasis may lead to gynecological disorders. MicroRNAs (miRNAs) are involved in a wide variety of physiological mechanisms and their expression patterns in the endometrium are currently attracting a lot of interest. miRNA regulation could be hormone dependent. Conversely, miRNAs could regulate the action of sexual hormones. Modifications to miRNA expression in pathological situations could either be a cause or a result of the existing pathology. The complexity of miRNA actions and the diversity of signaling pathways controlled by numerous miRNAs require rigorous analysis and findings need to be interpreted with caution. Alteration of miRNA expression in women with endometriosis has been reported. Thus, a potential diagnostic test supported by a specific miRNA signature could contribute to early diagnosis and a change in the therapeutic paradigm. Similarly, specific miRNA profile signatures are expected for RIF and endometrial cancer, with direct implications for associated therapies for RIF and adjuvant therapies for endometrial cancer. Advances in targeted therapies based on the regulation of miRNA expression are under evaluation.

## 1. Introduction

The human endometrium is the only tissue that undergoes cyclic monthly structural modifications including proliferation, differentiation and shedding of the superficial layer during the so-called menstrual phase [1]. During the proliferative phase, from day 1 of menses to ovulation day, the superficial layer regenerates from the basal layer under the action of estradiol. In the secretory phase, progesterone induces decidualization in the estradiol-primed endometrium, a crucial step for embryo implantation [2,3]. These structural and functional modifications at the cellular and intercellular levels are finely orchestrated by numerous extrinsic and intrinsic factors. The molecular responses to hormonal stimuli are modulated at the transcriptional and post-transcriptional stages. Imbalance in cellular and molecular endometrial homeostasis may lead to gynecological disorders such as endometriosis, implantation failure, and endometrial cancer [4,5,6,7,8].

MicroRNAs (miRNAs) are non-coding small RNAs composed of about 21–25 nucleotides. They modify the expression of around 60% of proteins at a post-transcriptional level. To date, 3000 miRNAs have been identified. A single miRNA can control the expression of several messenger RNAs (mRNAs) and a single mRNA may be targeted by more than one miRNA, thus creating a complex network of cooperative regulation [9].

miRNAs are involved in a broad variety of physiological mechanisms such as morphogenesis, differentiation, apoptosis, and cellular metabolism [10]. Expression levels of some miRNAs are associated with endometrial receptivity [11] and fluctuate according to progesterone blood levels during controlled ovarian hyperstimulation [12]. Their implication in the pathogenesis of endometriosis and endometrial cancer has been described [13,14]. Recently, we have shown that some miRNA expression profiles are associated with the prognosis of endometrial cancer [15].

## 2. Methodology

We identified relevant original studies in English language through a search of the MEDLINE (2010 to present) database using the following terms: “miRNA”, “miR”, “endometrium”, “recurrent implantation failure”, “endometriosis”, “endometrial cancer”. A first selection was made by eliminating redundant articles and retaining the most recent publication. Studies in languages other than English, review articles, meeting abstracts and posters were excluded.

## 3. miRNA Biogenesis and Function

miRNAs are non-coding RNAs composed of about 22 nucleotides. In animals, they are involved in the post-transcriptional regulation of protein expression. While they can be encoded by solitary genes controlled by their own promoters and regulatory sequences, most of the animal miRNAs are organized in tandem and arranged in clusters. Thus, they are frequently co-regulated together with other members of the cluster [16].

miRNAs are transcribed from introns of protein-coding genes or from the intergenic regions mainly by RNA polymerase II and, less frequently, by RNA polymerase III [17,18]. The primary miRNAs are then cleaved into stem-loop pre-miRNAs about 60–70-nt-long either by Drosha RNase III endonuclease or by an alternative Drosha-independent mirtron pathway. Pre-miRNAs are actively exported to the cell cytoplasm and cleaved by the Dicer enzyme, which removes the loop structure to form mature miRNA duplexes with an overhang at the newly formed 3′ end. Dicer guides the so-formed miRNA duplexes to the Argonaute (AGO) protein—part of the RNA-induced silencing complex (RISC)—which unwinds the duplexes to form single-stranded miRNA-5p and miRNA-3p products. The mature miRNA is integrated into RISC. Target mRNAs are bound to the miRNA–RISC complex and are thus deactivated as the ribosomal assembly is repressed. Bound mRNAs are stored in cytoplasmic structures called P-bodies, from which they are released upon a cellular signal or destroyed [19,20].

The endometrial expression of miRNAs is cell-dependent and their biogenesis is finely regulated by the physiopathological profile of the cell, the microenvironment, and environmental factors. Expression is dependent on a single nucleotide polymorphism, and epigenetic modifications regulate transcription (methylation or acetylation of DNA, histone modifications) and interactions with RNA-binding proteins, and edit miRNA maturation [20].

miRNAs are characterized by an imprecise complementarity to their target mRNA [16]. According to the degree of complementarity, the target mRNA can either be degraded or its translation blocked. The action of miRNAs also varies according to the target mRNA binding site and can even, in some conditions, activate gene expression [20].

In addition to this complexity of miRNA action, a single miRNA can target numerous mRNAs, thus regulating hundreds of proteins and various networks [21]. On the other hand, one mRNA can also be targeted by different miRNAs [20].

## 4. miRNA Detection Methods

miRNA can be isolated from a wide variety of tissues and body fluids [22]. Their quality and quantity accessible for analysis depend on the tissue/fluid conservation methods and extraction protocols [22].

Several miRNA detection techniques are available, including electrochemical or potentiometric technologies, Northern blotting techniques, surface plasmon resonance, next-generation sequencing (NGS), microarrays, real-time quantitative PCR (RT-qPCR), NanoString^®^ or immunoassays. All those technologies have advantages and drawbacks, with variable sensitivity and specificity, which could be a source of analytical bias and limit their utilization in routine practice [22]. Their principal characteristics are described in Table 1.

## 5. miRNAs and the Endometrial Cycle

### 5.1. The Endometrial Cycle

During the proliferative phase, a progressive proliferative growth of the endometrium is observed leading to a relative hypertrophy. This regular regeneration of the endometrial functional layer is a result of the proliferation and differentiation of epithelial and stromal stem/progenitor cells [23]. Numerous structural and molecular changes are brought about by estrogens. The functional layer of the endometrium is characterized by higher levels of expression of nuclear estrogen (ER) and progesterone receptors (PR) in both the epithelial and stromal cells in parallel with higher estrogen blood levels [1].

On a molecular level, the control of endometrial proliferation is highly complex. Estradiol, the key hormone during this endometrial phase, regulates gene expression at the transcriptional level via its nuclear receptors ERα and ERβ.

After ovulation, under the action of progesterone, the estrogen-primed endometrium undergoes a secretory differentiation called decidualization characterized by morphological and molecular modifications of perivascular stromal cells and increased angiogenesis. This includes an afflux of immune cells, vascular remodeling, as well as proliferation and differentiation of stromal fibroblasts and glandular epithelial cells [24]. The endometrial stromal cells are characterized by an absence of expression of nuclear ER and an increased expression of PR [1]. Progesterone plays the main role in the secretory endometrial phase by inhibiting the estrogen-regulated pathways involved in cell proliferation and the cell cycle. Conversely, it induces transcription factors regulating proapoptotic and cell cycle pathways [25]. Its functions are mediated by two main nuclear progesterone receptor isoforms—PR-A and PR-B—which share a common structure [26]. However, the increase in the relative expression of PR-A to PR-B might inhibit progesterone signaling [27].

### 5.2. Endometrial miRNA Regulation by Estrogen and Progesterone

The regulation of miRNA action by sexual hormones is possibly connected to the modulation of Dicer and AGO activities [19].

The impact of steroid sexual hormones on the expression of miRNA has been studied in animal models. However, studies in zebrafish showed that estrogen-regulated expression of miRNA is both cell- and tissue-specific [28]. Thus, miRNA hormonal regulation findings cannot necessarily be extrapolated to other species or tissues.

Exposure of estradiol to human endometrial stromal cells in an in vitro culture induced up-regulation of miR-181b and let-7e, and down-regulation of mi-R27b [29]. Similarly, induction of miR-125b and miR-133a expression was observed in an in vitro culture of human endometrial epithelial cells exposed to progesterone [30,31]. The induction of miR-133a expression resulted in a proliferation of endometrial epithelial cells [31].

In vivo, the endometrial expression of miR-30b, miR-125b, miR-424, and miR-451 was found to be lower in women with high levels of blood progesterone, compared with women with low progesterone blood levels, at the moment of the ovulation trigger during controlled ovarian hyperstimulation in an in vitro fertilization process [12].

Kuokkanen et al. [32] identified 49 miRNAs differentially expressed in the mid-secretory phase compared with the late proliferative phase in epithelial cells. Among the up-regulated miRNAs in the mid-secretory phase (compared with the late proliferative phase), miR-29b, miR-29c, miR-30b, miR-30d, miR-31, miR-193a-3p, miR-200c, miR-203, miR-204, miR-210, miR-345 and miR-582-5p showed the greatest differential expression levels. Conversely, the greatest differential expression levels among the down-regulated miRNAs in the late secretory phase were found for miR-105, miR-127, miR-134, miR-214, miR-222, miR-369-5p, miR-370, miR-376a, miR-382, miR450, miR-503 and miR-542-3p. In parallel, in the same study, transcriptomic analysis of the samples confirmed a down-regulation of 19 mRNAs in the mid-secretory phase predicted to be controlled by the identified miRNA.

Generally speaking, the pro-proliferative estrogen action on the miRNA profile is characterized by the inhibition of tumor suppressor miRNAs and induction of oncogene miRNA [33].

### 5.3. Endometrial Estrogen and Progesterone Action Regulation by miRNA

In the human endometrium, the action of estrogen is regulated by modifications in the expression of its nuclear receptors ERα and ERβ. The action of progesterone is dependent on the relative expression of the receptor isoforms PR-A and PR-B.

Little is known about miRNA regulation of ER in the endometrium. Bao et al. demonstrated that transfection of endometrial cancer cell lines with an miR-107 mimic resulted in a decreased expression of ERα at both the mRNA and protein levels. Inversely, transfection of the same cell line with an miR-107 inhibitor resulted in an increased expression of ERα at both levels [34]. Xiao et al. recently showed the impact of miR-22-5p on ERβ in endometrial stromal cells isolated from the eutopic endometrium of women with mild/severe endometriosis [35]. The transfection of these cells in an in vitro culture resulted in modifications of ERβ expression [35].

The expression of PR seems to be regulated by miR-194-3p, as the transfection of endometrial stromal cells with this miRNA in an in vitro culture resulted in a significant decrease in PR-A and PR-B protein expression [36]. In line with this finding, overexpression of miR-194-3p was correlated with the inhibition of endometrial stromal cell decidualization [36]. The same role has been described for miR-196a, as the transfection of endometrial stromal cells with this miRNA in an in vitro culture of endometrial cells resulted in decreased protein levels of PR-A and PR-B [37].

In in vitro studies, miR-92a promoted the proliferation of stromal endometrial cell lines. The transfection of this cell line with miR-92a resulted in progesterone resistance [38].

The functional analysis of miRNA showed that PRs are the predicted targets of miR-196a, miR-297, miR-575, miR-628–3p, miR-635, miR-921, miR-938 and miR-1184 [37].

## 6. miRNA and Embryo Implantation

Embryo implantation is a complex process during which a dialogue between the endometrium and the embryo is established. It takes place during a brief period of 4 days—called the receptive phase or the implantation window [2]—and is characterized by progesterone-induced immune tolerance, allowing embryo apposition, adhesion and invasion [1].

The success of embryo implantation is dependent on embryo quality, endometrial receptivity, and embryo–maternal interactions. The balanced expression of multiple molecules such as cytokines, chemokines, growth factors, lipids, and receptors is guaranteed by a fine autocrine, paracrine and juxtacrine regulation [39].

The presence of miRNAs in the uterine luminal fluid and their secretion in culture media suggest that they play a role in the implantation process. miRNAs seem to regulate uterine function as well as embryo development.

In mice, miR-223-3p is responsible for the reduction in pinopode formation [40] and, together with miR-181, is responsible for the down-regulation of leukemia inhibitory factor (LIF) expression [40,41]. Thus, the decreased expression of these two miRNAs is essential for initiating implantation [39].

Embryo apposition and adhesion require adhesive modifications in the apical surface of endometrial epithelium. The overexpression of Mucin-1, a transmembrane glycoprotein acting as an embryo attachment inhibitor, is related to embryo attachment failure. The expression of this protein in mice is probably regulated by miR-199a, let-7a and let-7b [42,43]. Conversely, type-1 insulin-like growth factor receptor (IGF1R), regulated by miR-145, promotes adhesive interactions [44].

Trophoblastic invasion is facilitated by epithelial modifications reflecting an epithelial–mesenchymal transition (EMT) with a decrease in cell polarization and remodeling of cell junctions. In a mouse model, miR-429—an miRNA involved in EMT regulation—has been shown to be down-regulated during implantation and its up-regulation resulted in a reduction in implantation sites by targeting protocadherin 8 [45]. On the other hand, miR-126-3p, which regulates integrin-α11 expression, and thus, modifies cell migration and invasion, is up-regulated in implantation sites [46]. miR-96 is also up-regulated in the implantation sites in early mouse pregnancy. It modulates the expression of anti-apoptotic factor Bcl-2 [47], which is involved in the decidualization process.

It has been suggested that miR-152-3p plays a role in embryo implantation and in the first days of pregnancy in the mouse. In epithelial endometrial cells, the expression of this miRNA, which is induced by progesterone, regulates the expression of GLUT3, which is involved in the regulation of intrauterine glucose concentration [48]. 

In in vitro cultures of human endometrial epithelial cells, increased miR-125b expression down-regulates the expression of matrix metalloproteinase-26 (MMP26), thus inhibiting cell movement and blocking embryo implantation [30]. On the other hand, in human endometrial stromal cells, miR-181a induces morphological transformation and regulates the expression of genes such as FOXO1A, PRL, IGFBP-1, DCN and TIMP3, which are involved in decidualization [49], and miR-222 modifies the expression of CDKN1C/p57kip2, which is involved in endometrial stromal cell decidualization and differentiation [50]. 

The expression of genes involved in endometrial maturation and its cyclic remodeling—such as CAST, CFTR, FGFR2, and LIF—are regulated by several miRNAs whose expression fluctuates according to the endometrial phase. miR-30b and miR-30d have been shown to be up-regulated, and miRNA-494 and miRNA-923 to be down-regulated in the receptive endometrium compared with the pre-receptive endometrium in healthy women [51].

miR-30b, miR-30d, miR-31 and miR-203 were found to be up-regulated during the implantation window compared with the proliferative phase [52]. The same study showed a down-regulation of miR-135a, miR-135b, miR-145 and miR-503.

The expression profiles of let-7, miR-17, the miR-30 family, miR-92 and miR-200 are associated with endometrial receptivity [11].

## 7. miRNA in Endometrial Disorders

Endometrial disorders associated with implantation failures, endometriosis and endometrial cancer are thought to arise from abnormal cellular and molecular endometrial homeostasis regulated at numerous stages of local gene expression.

### 7.1. Recurrent Embryo Implantation Failures (RIF)

RIF is defined as the absence of pregnancy after failure of implantation of at least three good-quality embryos [53] and is observed in up to 50% of couples undergoing assisted reproductive technology treatments. Identified causes of RIF are uterine abnormalities, spermatic factors, genetic abnormalities, hormonal and metabolic pathologies, thrombophilia, autoimmune diseases and excessive activation of the uterine immune profile [54,55].

At the molecular level, the expression of miR-145, which targets the mRNA coding for IGF1R [44], has been found to be up-regulated in women with RIF compared with fertile controls [56]. This study also showed up-regulation of nine other miRNAs (miR-23b, miR-27b, miR-99a, miR139-5p, miR-150, miR-195, miR-342-3p, miR-374b and miR-652) and down-regulation of three (miR-32, miR-628-5p and miR 874) in women with RIF [56]. The predicted target mRNA of the identified miRNAs is involved in adherent junction formation, Wnt signaling, cell adhesion, and the cell cycle. The predictive targets of miR-145—N-cadherin and netrin-4 (adhesion molecules)—were found to be down-regulated in women with RIF compared with fertile controls [56]. Similarly, H2AFX (involved in cell cycle arrest) and sFRP-4 (a Wnt signaling modulator), which are miR-23b targets, were also found to be down-regulated in women with RIF [56].

Another study of miRNA expression in the endometrium of women with RIF found up-regulation of six miRNAs (miR-29b-1-5p, miR-34b-3p, miR-138-1-3p, miR-146a-5p and miR-363-3p) [57].

The analysis of endometrial miRNA expression in women with ≥2 unsuccessful implantations compared to women with successful implantation after the first IVF attempt showed the up-regulation of miR-20b-5p, miR-155-5p and miR-330-5p and the down-regulation of miR-144-3p, miR-718 and miR-940 in women with RIF [58].

These miRNA modifications are summarized in Table 2.

### 7.2. Endometriosis

Endometriosis is a frequent gynecological condition where endometrium-like tissue is observed outside the uterus [62]. While the pathophysiological mechanism of endometriosis is not entirely clear, it is generally accepted that retrograde flux is implicated. However, changes in retrograde flux are frequently observed in the general population, including endometriosis-free women. Thus, other susceptibility factors play a role in the development and progression of endometriosis. Immunological and hormonal factors have been widely investigated [62]. The complex intracellular interactions between signaling pathways suggest that miRNAs may be implicated in the pathophysiology of endometriosis (Table 3) as they regulate numerous processes such as apoptosis and proliferation [63].

In the secretory phase of the eutopic endometrium in women with endometriosis, the miR-9 family and miR-34 are found to be down-regulated compared with healthy women [64]. The predicted target of miR-9 is Bcl-2, which is an anti-apoptotic protein whose expression is up-regulated in women with endometriosis [65].

The analysis of eutopic and ectopic endometrium in women with endometriosis found that miR-30c was down-regulated compared with the endometrium of healthy women [66]. This down-regulation was associated with an increased expression of plasminogen activator inhibitor type 1 (PAI-1). Transfection with this miRNA of endometrial stromal cells in an in vitro culture resulted in the down-regulation of PAI-1 and diminished cell proliferation, migration and invasion capacities [66].

Another study identified miR-196a as an overexpressed miRNA in the eutopic endometrium of women with endometriosis compared with healthy women [37]. miR-196a is involved in the regulation of the MEK/ERK signaling pathway [37]. Conversely, miR-451 was found to be down-regulated in the eutopic endometrium from women with endometriosis compared with healthy controls [67]. This down-regulation could be responsible for the promotion of proliferation and inhibition of apoptosis of endometrial cells [67].

Analysis of mesenchymal stem cells from the eutopic endometrium showed up-regulation of miR-200b and down-regulation of miR-145 and let-7b compared with mesenchymal stem cells from the endometrium of healthy controls [68]. Let-7 could have a regulatory impact on the expression of the genes involved in the maintenance of pluripotency of stem cells, their differentiation and self-renewal [69].

In women with progesterone-resistant endometriosis, defined as women without pelvic pain relief after progesterone treatment, miR-92a was found over-expressed in the eutopic endometrium compared with women with progesterone responsive endometriosis [38]. This over-expression was related to a decreased expression of PTEN, which is involved in the repression of cellular division and promotion of apoptosis [70].

Among the deregulated miRNAs in the ectopic endometrium of women with endometriosis compared with eutopic endometrium, 14 have been found to be up-regulated (miR-1, miR-29c, miR-99a, miR-99b, miR-100, miR-125a, miR-125b, miR-126, miR-143, miR-145, miR-150, miR-194, miR-223 and miR-365), and 8 down-regulated miRNA (miR-20a, miR-34c, miR-141, miR-142-3p, miR-196b, miR-200a, miR-200b and miR-424) [71].

The down-regulation of miR-34a-5p in ectopic endometrial tissue was correlated with the expression of VEGFA [60]. The regulation of this protein expression by miR-34a-5p was confirmed by the transfection of endometrial stromal cells with this miRNA in an in vitro culture, which resulted in down-regulation of VEGFA expression and diminution of the cell proliferation [60].

Analysis of ectopic endometrium found a down-regulation of miR-141-3p compared with eutopic endometrium and to endometrium from healthy controls [49]. This miRNA seems to be involved in the regulation of apoptotic factors, as its down-regulation resulted in elevated Bcl-2 expression and decreased Bax expression, thus inhibiting apoptosis [49].

It has been suggested that down-regulation of miR-200b and miR-200c is involved in the cell proliferation, migration and invasion capacities of endometrial cells [72,73]. Their action could be associated with EMT, which is regulated by ZEB1 and ZEB2 [72,73].

Zhou et al. identified miR-205-5p as being down-regulated in the ectopic endometrium of women with endometriosis compared with the normal endometrium of women with cervical epithelial cancer or leiomyomas [74]. In an in vitro culture of endometrial stromal cells, the authors demonstrated suppressed migration and invasion, and promotion of apoptosis after transfecting the cells with miR-205-5p. It has been suggested that angiopoietin-2 (ANGPT2) is the downstream mediator of this miRNA action [74]. miR-210-3p is also thought to be involved in the development of endometriosis as this miRNA was found to be up-regulated in both the eutopic and ectopic endometrium of women with endometriosis compared with the endometrium of healthy women [75]. This miRNA could be involved in cell proliferation and the DNA damage response to oxidative stress [75]. The role of miR-33b in endometrial proliferation regulation has also been suggested [76]: its expression is down-regulated in the ectopic endometrium compared to normal endometrial tissue. Transfection of endometrial cells with an miR-33b inhibitor in an in vitro culture resulted in increased cell proliferation. This was associated with a decreased expression of Caspase-3 and increased expression of VEGF and MMP-9 [76].

The association between endometriosis and infertility is not completely understood. During the implantation window, miR-543 was found to be down-regulated in the eutopic endometrium of women with endometriosis compared with healthy controls [77]. In the normal endometrium, expression of miR-543 increases from the proliferative phase to the implantation window, which suggests that it may be involved in the implantation process, thus explaining the potential mechanism of fertility impairment in women with endometriosis [77].

Analysis of the eutopic endometrium from the mid-secretory phase in women with endometriosis showed an over-expression of miR-194-3p in endometrial stromal cells compared with women without endometriosis [36]. In a mouse model, this miRNA was found to be involved in the regulation of the STAT1/mTOR signaling pathway and its down-regulation in ectopic endometrial epithelial cells promoted cell proliferation and invasion [78]. In the eutopic endometrium of women with mild or minimal endometriosis, miR-194-3p was found to regulate PR expression and to increase the PR-A/PR-B mRNA ratio, thus impairing decidualization [36].

Analysis of ectopic stromal cells compared with the eutopic endometrium of women with endometriosis showed up-regulation of miR-139-5p, which can have a suppressive impact on the expression of HOXA10 [61], a gene involved in embryo implantation. Joshi et al. demonstrated a significant up-regulation of miR-29c in the mid-secretory phase in the eutopic endometrium of women with endometriosis compared with healthy controls [79]. Transfection with miR-29c was associated with a decreased expression of FKBP4 and decreased decidualization response to progesterone in an in vitro culture of decidual cells [79]. Moreover, the authors showed that the excision of endometriotic lesions decreased the expression of miR-29c.

Hawkins et al. identified 10 up-regulated miRNAs (miR-29c, miR-100, miR-193a-3p, miR-193a-5p, miR-202, miR-485-3p, 509-3-5p, 574-3p, miR-708 and miR-720) and 12 down-regulated (miR-10a, miR-34c-5p, miR-141, miR-200a, miR-200b, miR-200c, miR-203, miR-375, miR-429, 449b, miR-504 and miR-873) in endometriomas compared with eutopic endometrium [80].

**Table 3:** Hormonal regulation and function of deregulated miRNAs in endometriosis.

miRNA	Sex Steroid Hormone Regulation in Endometrium	Function	Endometriosis
Let-7 family		Maintenance of pluripotency of stem cells, their differentiation and self-renewal [60]	
Let-7b		Regulation of Mucin-1 expression in mouse	Down-regulated in mesenchymal stem cells from eutopic endometrium [59]
Let-7c-5p			Up-regulated in endometriosis-associated infertility [29]
miR-1			Up-regulated in ectopic endometrium [62]
miR-9		Regulation of Bcl-2 expression (apoptosis) [56]	Down-regulated in secretory phase of eutopic endometrium [55]
miR-20a			Down-regulated in ectopic endometrium [62]
miR-22-5p			Down-regulated in endometriosis-associated infertility [29]
miR-26b	Up-regulated in implantation window compared with pre-receptive endometrium [43]		(miR-26b-3p) Down-regulated in endometriosis-associated infertility [29]
miR-29c	Up-regulated in secretory phase [24]	Regulation of FKBP4 and decidualization response to progesterone [70]	Up-regulated in ectopic endometrium [62,70]
miR-30c		Regulation of PAI-1 [57]	Down-regulated in eutopic and ectopic endometrium [57]
miR-33b		Regulation of VEGF, MMP-9 and Caspase-3 and endometrium proliferation [67]	Down-regulated in ectopic endometrial tissue [67]
miR-34			Down-regulated in secretory phase of eutopic endometrium [55]
miR-34a-5p		Regulation of VEGFA expression and cell proliferation [51]	Down-regulated in ectopic endometrium [51]
miR-34c			Down-regulated in ectopic endometrium [62]
miR-92a		Regulation of PTEN (repression of cellular division and promotion of apoptosis) [61]	Up-regulated in eutopic endometrium in progesterone resistant endometriosis [30]
miR-99a			Up-regulated in ectopic endometrium [62]
miR-99b			Up-regulated in ectopic endometrium [62]
miR-100			Up-regulated in ectopic endometrium [62]
miR-125a			Up-regulated in ectopic endometrium [62]
miR-125b	Up-regulated by progesterone in vitro [22,23]; Down-regulated by progesterone if high progesterone blood level [7]	Inhibition of cell movement and blocking embryo implantation via regulation of MMP26 in vitro	Up-regulated in ectopic endometrium [62]
miR-126	up-regulated in implantation sites in mouse [38]	Regulation of integrin-α11 expression	Up-regulated in ectopic endometrium [62]
miR-139-5p		Regulation of *HOXA10* expression [52]	Up-regulated in ectopic stromal cells [52]
miR-141			Down-regulated in ectopic endometrium [62]
miR-141-3p		Regulation of apoptotic factors [41]	Down-regulated in ectopic endometrium [41]
miR-142-3p			Down-regulated in ectopic endometrium [62]
miR-143			Up-regulated in ectopic endometrium [62]
miR-145		Regulation of IGF1R expression in mouse Down-regulation of N-cadherin and netrin-4 (adhesion molecules) in RIF	Up-regulated in ectopic endometrium [62] Down-regulated in mesenchymal stem cells from eutopic endometrium [59]
miR-150			Up-regulated in ectopic endometrium [62]
miR-194			Up-regulated in ectopic endometrium [62]
miR-194-3p	Regulation of PR-A/PR-B ratio in eutopic endometrium in endometriosis [28]	Regulation of STAT1/mTOR signaling pathway [69]	Up-regulated in mid-secretory phase of eutopic endometrium [28]
miR-196a		MEK/ERK signaling pathway [29]	Up-regulated in eutopic endometrium [29]
miR-196b			Down-regulated in ectopic endometrium [62]
miR-199a		Regulation of Mucin-1 expression in mouse	Up-regulated in eutopic endometrium
miR-200a			Down-regulated in ectopic endometrium [62]
miR-200b		Regulation of EMT via *ZEB1* and *ZEB2* [63]	Down-regulated in ectopic endometrium [62,63] Up-regulated in mesenchymal stem cells from eutopic endometrium [59]
miR-200c	Up-regulated in secretory phase [24]	Regulation of EMT via *ZEB1*, *ZEB2* and *MALAT1* [64]	Down-regulated in ectopic endometrium [64]
miR-205-5p		Regulation of migration, invasion and apoptosis via ANGPT2 [65]	Down-regulated in ectopic endometrium [65]
miR-210-3p	Up-regulated in secretory phase [24]	Regulation of cell proliferation and DNA damage response to oxidative stress [66]	up-regulated in eutopic and ectopic endometrium [66]
miR-223			Up-regulated in ectopic endometrium [62]
miR-365			Up-regulated in ectopic endometrium [62]
miR-424	Down-regulated by progesterone if high progesterone blood level [7]		Down-regulated in ectopic endometrium [62]
miR-451		Regulation of proliferation and apoptosis [58]	Down-regulated in eutopic endometrium [58]
miR-543			Down-regulated in eutopic endometrium during implantation window [68]

### 7.3. Endometrial Cancer

Endometrial cancer is the first most common gynecological cancer in developed countries, with 380,000 new cases diagnosed each year worldwide [81].

miRNA expression has been widely investigated in endometrial cancer. The pattern of their expression is associated with prognostic factors such as lymph node involvement, lymphovascular space invasion, overall survival and recurrence-free survival [82]. In a recent meta-analysis, Delangle et al. identified 54 over-expressed and 59 down-regulated miRNAs in tumoral endometrial tissue compared with healthy endometrium [82].

EMT is one of principal processes involved in cancer invasion and metastasis and is characterized by the phenotypical, morphological and molecular changes of cancer cells, resulting in increased mobility, loss of polarity, and decreased properties of cellular adhesion [83]. Numerous miRNAs have been implicated in the process of EMT in the literature (Table 4). In endometrial cancer, the up-regulation of miR-141, miR-200, miR-203, miR-205 and miR-429 and the down-regulation of miR-133 and miR-224 seem to be a molecular signature of EMT in endometrial cancer [84]. The let-7 family has been found to be involved in the promotion of EMT in endometrial carcinosarcomas [85]. The action of miR-141 and miR-200 is thought to be based on the regulation of ZEB1/ZEB2 expression involved in the regulation of E-cadherin expression [86,87,88]. Let-7 is thought to regulate the expression of the high mobility group AT-hook 2 (HMGA2), an embryonic nuclear factor [85], which in turn regulates the expression of Snail, Slug, ZEB1, and ZEB2 inducing EMT [89].

Analysis of endometrial serous adenocarcinoma showed an up-regulation of miR-205 and down-regulation of miR-10b, miR-29b, miR-34b, miR-101, miR-133a, miR-133b, miR-152 and miR-411 compared with normal endometrium [90]. The expression of miR-10b and miR-29b was correlated with vascular invasion [90].

In endometrioid endometrial cancer, analysis of the miRNA endometrial profile showed the up-regulation of miR-106a and miR-144 and the down-regulation of miR-30d compared to controls [91]. Those miRNA seem implicated in the expression regulation of genes involved in EMT [91].

## 8. Perspectives

Cumulative data demonstrate that miRNAs are involved in various gynecological disorders, including endometriosis, with potential diagnostic and therapeutic implications. Thus, diagnostic tests supported by a specific miRNA signature according to the disorder could be developed. This could contribute to an early diagnosis for women with endometriosis and a subsequent change in the therapeutic paradigm. Similarly, such a diagnostic test could determine associated therapies for women with RIF, and adjuvant therapies for endometrial cancer. Advances in targeted therapies based on the regulation of miRNA expression are under evaluation.

## 9. Conclusions

miRNA are non-coding small RNAs responsible for the post-transcriptional regulation of gene expression. A single miRNA can control the expression of several mRNAs and a single mRNA may be targeted by more than one miRNA, thus creating a complex network of cooperative regulation. The action of each miRNA is tissue-specific and cannot be generalized to other organs.

miRNA expression patterns in the physiological and pathological endometrium are currently a hot topic of research. miRNA expression profile in the human endometrium varies according to the menstrual cycle and is regulated by sex steroids. On the other hand, miRNA can impact the action of sex steroids, so that modifications in miRNA expression profile in pathological endometrium could either be a cause or a result of the pathology. 

Deregulated miRNA identified in RIF patients are frequently involved in adhesion, proliferation, and angiogenesis processes. In endometriosis the identified miRNA are frequently associated to proliferation, apoptosis and cell adhesion. Finally, miRNA involved in EMT are frequently found in endometrial cancer.

Recent progress allowing global analysis of known miRNA in the human endometrium may allow further understanding of their actions and hopefully lead to the development of diagnostic and/or theragnostic signatures. 

## Figures and Tables

**Table 1:** Characteristics of miRNA detection methods (adapted from Kappel and Keller, 2017 [22]).

	Application	Sensitivity	Specificity	Availability
Electrochemical or potentiometric	Diagnostic test	High	Excellent	Limited
Northern blotting	Validation technique	Poor	High	Widely available
NGS	Discovery, validation, diagnostic	High	High	Limited
Microarray	Discovery, validation, diagnostic	Low	Low	Rather available
RT-qPCR	Validation, diagnostic	Excellent	Excellent	Widely available
NanoString^®^	Validation, diagnostic	High	High	Limited
Immunoassay	Diagnostic test	Low	Low	Limited

**Table 2:** Hormonal regulation and function of deregulated miRNAs in recurrent implantation failure.

miRNA	Sex Steroid Hormone Regulation in Endometrium	Function in the Endometrium	RIF
miR-20b-5p			Up-regulated [58]
miR-21 miR-21-3p miR-21-5p	Up-regulated in implantation window compared with pre-receptive endometrium [51]		Down-regulated [57] Up-regulated [59]
miR-23b		Regulation of H2AFX (involved in cell cycle arrest) and sFRP-4 (Wnt signaling modulator) expression [56]	Up-regulated [56]
miR-27b	Down-regulated by estrogen in vitro [29]		Up-regulated [56]
miR-29b	Up-regulated in secretory phase [32]		Up-regulated [57]
miR-32			Down-regulated [56]
miR-34 miR-34a-5p miR-34b		Regulation of VEGFA expression and cell proliferation [60]	Up-regulated [57]
miR-99a			Up-regulated [56]
miR-138-1-3p			Up-regulated [57]
miR-139-5p		Regulation of *HOXA10* expression [61]	Up-regulated [56]
miR-144-3p			Down-regulated [58]
miR-145	Down-regulated in the implantation window [52]	Regulation of IGF1R expression in mouse [44]; down-regulation of N-cadherin and netrin-4 expression (adhesion molecules) in RIF [56]	Up-regulated [56]
miR-146a-5p			Up-regulated [57]
miR-150			Up-regulated [56]
miR-155-5p			Up-regulated [58]
miR-195			Up-regulated [56]
miR-330-5p			Up-regulated [58]
miR-342-3p			Up-regulated [56]
miR-363-3p			Up-regulated [57]
miR-374b			Up-regulated [56]
miR-628	Predicted target of progesterone [37]		Down-regulated [56]
miR-652			Up-regulated [56]
miR-718			Down-regulated [58]
miR-874			Down-regulated [56]
miR-940			Down-regulated [58]

**Table 4:** Hormonal regulation and function of deregulated miRNAs in endometrial cancer.

miRNA	Sex Steroid Hormone Regulation in Endometrium	Function	Endometrial Cancer
miR-10b		vascular invasion [90]	Down-regulated [90]
miR-29b	Up-regulated in secretory phase [32]	vascular invasion [90]	Down-regulated [90]
miR-30d		Promotion of EMT [91]	Down-regulated [91]
miR-34b			Down-regulated [90]
miR-101			Down-regulated [90]
miR-106		Promotion of EMT [91]	Up-regulated [91]
miR-133	Up-regulated by progesterone [31]	Promotion of EMT [84]; promotion of endometrial epithelial cell proliferation [31]	Down-regulated [90]
miR-141		Promotion of EMT [84]; regulation of ZEB1/ZEB2 expression [86,87,88]	Up-regulated [84,90]
miR-144		Promotion of EMT [91]	Up-regulated [91]
miR-152	Up-regulated by progesterone [48]	regulation of GLUT3 expression [48]	Down-regulated [90]
miR-200		Promotion of EMT [84]; regulation of ZEB1/ZEB2 expression [86,87,88]	Up-regulated [84]
miR-203	Up-regulated in secretory phase and in implantation window [32]	Promotion of EMT [84]	Up-regulated [84]
miR-205		Promotion of EMT [84]	Up-regulated [84,90]
miR-224		Promotion of EMT [84]	Down-regulated [84]
miR-411			Down-regulated [90]
miR-429	down-regulated during implantation in mouse [45]	Promotion of EMT [84]	Up-regulated [84]
